# Femoral neck fracture increases 30-day mortality 16-fold and elevates mortality risk for up to 4 years: a matched cohort study of 3,246 patients

**DOI:** 10.1007/s41999-026-01449-3

**Published:** 2026-03-28

**Authors:** Peter Larsen, Stefan Teglhus Jensen, Rasmus Elsoe

**Affiliations:** Department of Orthopaedic Trauma Surgery, Aalborg University Hospital, 18-22 Hobrovej, 9000 Aalborg, Denmark

**Keywords:** Femoral neck fracture, Mortality, Comorbidities, Match group design

## Abstract

**Methods:**

The study design was a matched cohort study based on prospectively collected registry data from the Danish National Patient Register. Included were all patients presenting with an FNF in the Northern Region of Denmark between 2018 and December 2023. The matched reference population was established with a 1:10 ratio (for each patient with an FNF, we included ten citizens who had not experienced an FNF prior to the inclusion date). Basic characteristics including age, gender, and individual comorbidity status were obtained from all patients and the matched population.

**Results:**

A total of 3246 patients were identified with an FNF during the 6-year study period (mean follow-up 2.6 years). Patients’ mean age at the time of fracture was 77.9 (95% CI 77.4–78.4). Male mean age was 74.9 years (95% CI 73.9–75.9). Female mean age was 79.6 years (95% CI 79.1–80.2). Gender distribution was 64% females and 36% males. A total of 761 (23.4%) patients died during the first 12 months after the fracture. Within the first 30 days after fracture, patients with FNFs demonstrated markedly increased mortality compared with the age-, sex-, and comorbidity-matched reference cohort, with a crude 30-day mortality of 10% versus 0.3% and a hazard ratio of 16.1. Cumulative survival curves showed that patients with FNFs had an elevated mortality risk during the first 4 years following fracture, after which survival became nearly comparable between the fracture and matched cohorts.

**Conclusion:**

This study demonstrated that FNFs are associated with a markedly elevated short-term mortality risk, with patients experiencing a 16 times higher risk of death within 30 days compared with an age-, gender-, and comorbidity-matched group without an FNF. The excess risk was most pronounced during the first 3–6 months and persisted for up to 4 years before converging with the match group mortality rates.

## Key summary points

**Aim:**

The primary aim of this study was to investigate the 30-day, 3-, 6-, and 12-month mortality between patients with femoral neck fractures (FNFs) and an age-, gender-, and comorbidity-matched population without a prior FNF. The secondary aim of this study was to report the long-term cumulative survival rate in patients with FNFs compared to that of an age-, gender-, and comorbidity-matched population without a prior FNF.

**Findings:**

FNFs are associated with a markedly increased mortality compared with the age-, sex-, and comorbidity-matched reference cohort, with a crude 30-day mortality of 10% versus 0.3% and a hazard ratio of 16.1. The excess risk was most pronounced during the first 3–6 months and persisted for up to 4 years before converging with the match group mortality.

**Message:**

Considering the matching on age, sex, and comorbidity status in the present study, the results suggest that factors not associated with patients’ baseline health status may contribute to the elevated risk of mortality among patients with FNFs. This study showed a 16 times elevated risk of mortality compared to the age-, gender-, and comorbidity-matched group of citizens without an FNF at 30 days.

## Introduction

The incidence of femoral neck fractures (FNFs) increases significantly after the age of 65 years and occur more common in women compared to men [1]. Recent European data report incidence rates to be 259/100,000 for males and 325/100,000 for females [2].

Patients sustaining FNFs often present with multiple comorbidities [3], and more than half have moderate to severe comorbidities [4]. Common comorbidities include anemia, hypertension, cardiovascular diseases, pulmonary diseases, diabetes, dementia, and osteoporosis [3].

The presence and severity of comorbidities are strong predictors of short-term mortality following FNFs [3, 5]. Specific comorbidities such as heart failure, stroke, dementia, and respiratory and renal diseases are strong predictors of higher mortality following FNFs [3]. In addition, social factors such as dependency and living in a nursing home are also reported with a strong association to increased mortality [6].

Although several studies have examined mortality rates, incidence of comorbidities, and the association between comorbidity and mortality in patients with FNFs, large-scale studies that match on age, gender, and comorbidity remain limited [3, 5, 7, 8]. Current evidence provides only limited information on how trauma, fracture, treatment, and preexisting comorbidities interact to influence mortality risk in patients with FNFs. FNFs represent a major traumatic event in frail elderly patients and may independently increase mortality risk regardless of comorbidity status. Although age and gender account for two key demographic factors strongly associated with mortality in FNFs, residual confounding related to baseline health remains a concern, as comorbidity is also a strong predictor of mortality [9, 10]. Incorporating comorbidity in addition to age and gender matching may, therefore, provide a more accurate understanding of the mortality risk in patients following FNFs [10].

The primary aim of this study was to investigate the 30-day, 3-, 6-, and 12-month mortality between patients with FNFs and an age-, gender-, and comorbidity-matched population without a prior FNF. The secondary aim of this study was to report the long-term cumulative survival rate in patients with FNFs compared to that of an age-, gender-, and comorbidity-matched population without a prior FNF. Furthermore, the potential interaction effects of individual comorbidities on mortality will be investigated.

## Methods

### Study design

The study design was a matched cohort study based on prospectively collected registry data from the Danish National Patient Register [11, 12].

Included were all patients presenting with an FNF in the Northern Region of Denmark between January 1, 2018 and December 31, 2023.

The Local Ethics Committee responded to this study request stating that the study design does not need notification. The study was approved by the Institutional Review Board (J. nr. 1-45-72-1-25). The reporting of the study complies with the Strengthening the Reporting of Observational Studies in Epidemiology (STROBE) statement [13].

### Study population and data retrieval

Patients registered with the ICD-10 diagnosis code for “femoral neck fracture” (DS720) were identified in the Danish National Patient Register. Excluded were patients with prior FNFs. To facilitate successful 1:10 matching on age, sex, and comorbidity, we included only patients with femoral neck fractures, which represent over 50% of hip fractures in the Danish population [14]. Focusing on FNFs allowed for a more homogeneous cohort and a more precise assessment of mortality risk within this fracture subtype.

Basic characteristics such as age, gender, and comorbidity status were obtained. Individual patients were monitored for death and emigration from the time of inclusion and until December 31, 2024.

The matched reference population was established with a 1:10 ratio (for each patient with an FNF, we included ten citizens who had not experienced an FNF prior to the inclusion date, if possible). The reference group was matched based on age, gender, and comorbidity status. Individual citizens in the match group were monitored for death and emigration from the time of inclusion (match time) until December 31, 2024.

Patients were censored in case of emigration from the country.

Data of comorbidities on an individual level for both the case and the match group were obtained through the Danish National Patient Register. Comorbidities were predefined using seven diagnostic categories: cancer, diabetes, pulmonary disease, stroke and dementia, liver disease, kidney disease, and cardiovascular disease. The seven predefined diagnostic categories were treated binary (yes/no). Comorbidity groups were established based on ICD-9 and ICD-10 diagnostic codes from January 1, 1971 until the date of fracture/inclusion (see Supplementary Table 1).

### Statistical methods

Mean values and 95% confidence intervals/ranges are given for continuous variables. Percentages and frequencies are given for categorical data.

The ratio (HR) for 30-day, 3-, 6-, and 12-month mortality was calculated using a Cox proportional hazards regression model. The cumulative survival rates were plotted using Kaplan–Meier analysis throughout the 6-year observational period. A Cox proportional hazards regression model was run to analyze the potential interaction effects of individual comorbidities on mortality. All analyses were performed using Stata statistical software (StataCorp LP).

## Results

A total of 3246 patients were identified with an FNF during the 6-year study period (mean follow-up 2.6 years). Patients’ mean age at the time of fracture was 77.9 (95% CI 77.4–78.4). Male mean age was 74.9 years (95% CI 73.9–75.9). Female mean age was 79.6 years (95% CI 79.1–80.2). Gender distribution was 64% females and 36% males. Baseline characteristics of the case and match group are presented in Table 1.

**Table 1:** Baseline characteristics

	Case group	Match group
Number of patients, N	3246	27597
Female, N (%)	2083(64%)	18121(66%)
Men, N (%)	1163(36%)	9476(34%)
Mean age at fracture/match, (95% CI)	77.9(77.4–78.4)	77.1(77.0–77.3)
Female	79.6(79.1–80.2)	79.1(78.9–79.3)
Men,	74.9(73.9–75.9)	73.4(73.1–73.8)
Mean follow-up time, years(95% CI)	2.6(2.5–2.6)	3.3(3.3–3.3)
*Comorbidities, N (%)*		
Cancer	735(23%)	5153(19%)
Pulmonary disease	580(18%)	3857(14%)
Cardiovascular disease	849(26%)	6161(22%)
Stroke/dementia	1059(33%)	7845(28%)
Kidney disease	309(10%)	1048(4%)
Liver disease	92(3%)	287(10%)
Diabetes	407(13%)	2241(8%)

*N* = number, *CI* = confidence interval

### Primary outcome

A total of 761 (23.4%) patients died during the first 12 months after the fracture. The mean age of deceased patients was 84.6 years (83.9–85.3) with 60.4% female.

Crude mortality rates and risk of mortality at 30 days, 3, 6, and 12 months are presented in Table 2 and 2a.

**Table 2:** Crude mortality rates

	Crude mortality rates %, (N)			
	30 days	3 months	6 months	12 months
Cases	10% (323)	15.0% (488)	18.7% (607)	23.4% (761)
Female	9.0% (188)	14.0% (292)	17.5% (364)	22.1% (461)
Male	11.6% (135)	16.9% (196)	20.6% (240)	25.8% (300)
Match group	0.3% (94)	1.1% (315)	2.2% (604)	4.2% (1160)
Female	0.4% (68)	1.2% (221)	2.3% (408)	4.3% (771)
Male	0.3% (26)	1.0% (94)	2.1% (199)	4.1% (389)

Table 2a: Risk (hazard rate)				
	Hazard ratio (95%CI)			
	30 days	3 months	6 months	12 months
Overall	16.1(12.5–20.8)	7.9(6.7–9.3)	5.4(4.8–6.2)	3.7(3.3–4.1)
Female	12.7(9.4–17.2)	6.7(5.5–8.2)	4.8(4.1–5.7)	3.3(2.9–3.8)
Male	26.2(16.2–42.2)	10.9(8.2–14.5)	6.7(5.4–8.4)	4.4(3.7–5.3)

*N* = number, 95%CI: 95% confidence interval

Within the first 30 days after fracture, patients with FNFs demonstrated markedly increased mortality compared with the age-, sex-, and comorbidity-matched reference cohort, with a crude 30-day mortality of 10% versus 0.3% and a hazard ratio of 16.1.

### Secondary outcomes

**Fig. 1:**
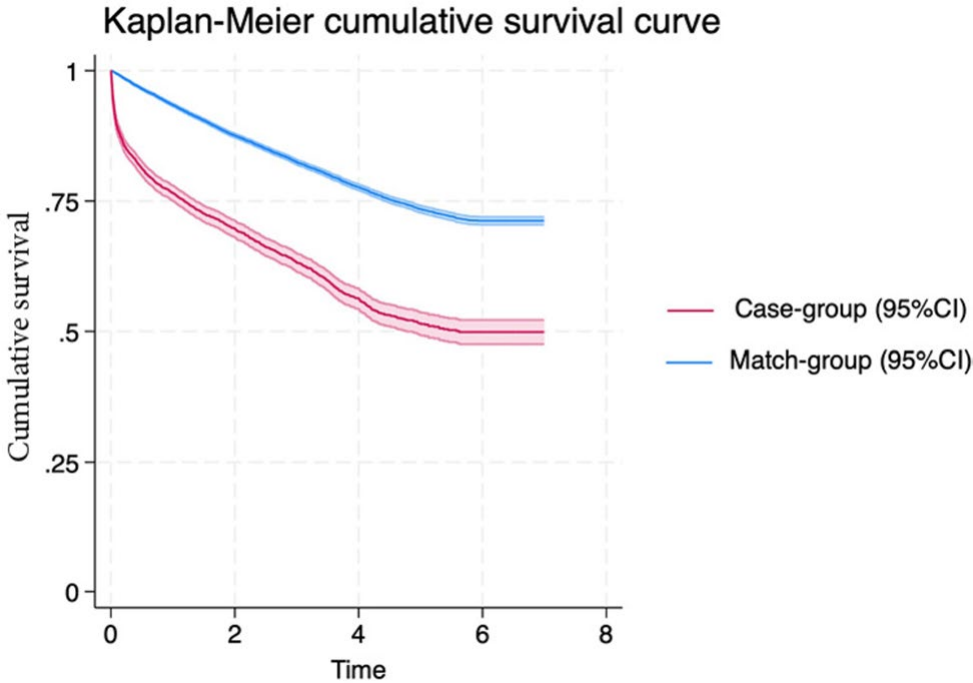
Kaplan-meier cumulative survival curve

Cumulative survival curves (Fig. 1) showed that patients with FNFs had an elevated mortality risk during the first 4 years following fracture, after which survival became nearly comparable between the fracture and matched cohorts. Results from the Cox proportional hazards model (HR 95% CI) at 0–1, 3–4, and 5–6 years were 3.7 (3.3–4.1), 2.9 (2.2–3.8), and 2.1 (1.0–4.7), respectively.

Results from the Cox proportional hazards model assessing potential interaction effects of individual comorbidities on mortality are shown in Table 3. No significant interaction between individual comorbidities and mortality was observed between the two cohorts. Results suggest that, within this analysis, the effects of individual comorbidities on mortality were not significantly modified by the presence of other comorbidities.

**Table 3:** Synergistic effects of individual comorbidities on mortality

	Hazard rate (95% CI)
Cancer	0.9(08–1.2)
Diabetes	1.0(0.8–1.4)
Stroke/dementia	1.1(0.9–1.3)
Cardiovascular disease	1.0(0.8–1.2)
Pulmonary disease	1.0(0.6–1.7)
Liver disease	2.3(0.9–5.6)
Kidney disease	1.3(0.9–1.9)

A higher risk of mortality with a HR of 2.4 (95% CI 2.1–2.8) was found in the case group compared to the match group when no comorbidities were present. When comparing patients with FNFs presenting with at least one comorbidity to patients without comorbidities, a higher risk of mortality was observed (HR 2.7, 95% CI 2.3–3.1). Comparable results were observed for the match group (HR 2.3, 95% CI 2.1–2.4).

## Discussion

The main results from the present study indicated that patients with FNFs were about 16 times (HR 16.1) more likely to die during the first 30 days after fracture compared to citizens presenting with comparable age, gender, and comorbidity status without an FNF. The crude 30-day mortality rate was 10% in the case group and 0.3% in the matched group.

This study is one of few to report mortality rates from a match design including age, gender, and individual comorbidities. Several studies examining outcomes in patients with FNFs have used matched designs, most commonly matching on age and sex, with only a few also incorporating comorbidity matching [9, 10]. While this approach takes into account two demographic factors with known strong associations to mortality in patients with FNFs, residual confounding by baseline health status remains a concern, as comorbidity is also strongly associated with increased mortality [10]. Matching on comorbidity in addition to age and gender may improve the understanding of mortality in FNFs. Incorporating comorbidity into the matching process enhances validity by ensuring greater comparability between groups, thereby allowing observed differences in mortality to be more confidently attributed to the fracture itself rather than to underlying comorbidity status.

The crude 30-day, 3-, 6-, and 12-month mortality rate in the FNF group was 10%, 15%, 19%, and 23%, respectively. The mortality rates in the match group without FNFs were 0.3%, 1%, 2%, and 4%, respectively. The observed mortality rate in patients with FNFs in the present study is considerable, although consistent with previous literature [3, 15, 16]. Female presented with a mortality risk of about 12 (HR 12.7), while male gender presented with a mortality risk of about 26 (HR 26.2), during the first 30 days after fracture compared to the match group. The observed higher mortality in male gender following FNFs is commonly reported in existing literature [17].

Considering the matching on age, sex, and comorbidity status in the present study, our results suggested that factors not associated with patients’ baseline health status may contribute to the elevated risk of mortality among patients with FNFs. This is supported by a more than two times higher risk of death (HR 2.4) when no comorbidities were present. However, the design of the present study implies that no conclusion regarding causality can be drawn.

Time to surgery, optimizing nutrition, comprehensive geriatric assessment, fast mobilization following surgery, and treatment in specialized FNF units are commonly reported to be associated with a decreased risk of mortality [18–21]. In Denmark, including our institution, time to surgery, optimizing nutrition, comprehensive geriatric assessment, fast mobilization following surgery, and treatment in specialized FNF units are monitored by a national reference program, the Danish Multidisciplinary Hip Fracture Registry (DMHFR) [22]. Despite this, our study showed a 16 times elevated risk of mortality compared to the age, gender, and comorbidity match group of citizens without an FNF at 30 days.

In comparison to Lunde et al.[10], matching on comorbidity using the Charlson Comorbidity Index reported substantially higher mortality in hip fracture patients compared with matched controls. Similarly, Vestergaard et al. [23] demonstrated that hip fracture is an important independent predictor of mortality, with excess risk beyond that conferred by preexisting comorbidities and post-fracture complications. In addition, a meta-analysis by Haentjens et al. [24] confirmed that hip fracture increases mortality even after accounting for age, sex, and comorbidities. Together, these studies support our observation that, when patients are matched on age, sex, and comorbidity, the occurrence of a femoral neck fracture remains associated with markedly increased mortality.

The cumulative survival of patients with and without FNFs throughout the 6-year observational time period showed an increased risk of death during the first 4 years after fracture, followed by an almost comparable risk of death between the case and match group throughout the remaining observational period. A particularly high risk was observed during the first 3–6 months after fracture. This trajectory is consistent with existing literature, commonly reporting pronounced excess mortality within the first year after FNFs, followed by a gradual convergence toward background mortality in subsequent years [25, 26].

As expected, a high degree of comorbidity was observed among patients with FNFs in the present study. Prevalence ranged from 3% for liver disease to 33% for stroke/dementia. The most frequent comorbidities were stroke/dementia (33%), cardiovascular disease (26%), and cancer (23%). Direct comparisons with the existing literature are limited by high heterogeneity in the classification and reporting of comorbidity status. Nonetheless, the prevalence estimates reported from the present study are broadly consistent with the existing literature and may mainly reflect patients’ relatively high age at the time of fracture (mean age 77.9) [3, 27, 28].

Comorbidity is well established as a predictor of increased mortality following FNFs [3]. However, the potential interaction effects of specific comorbidities on mortality in FNFs remain unclear. In the present study, no statistically significant interaction effects between individual comorbidities and mortality were observed when comparing the case and matched groups, suggesting that comorbidities may not confer a substantial additional combined effect on mortality in this cohort of FNF patients. Thus, while comorbidity remains an important risk factor for mortality, the effects of individual comorbidities appear to be largely additive with no clear evidence of interaction between comorbidities. These findings should be interpreted with caution, as the use of broad comorbidity categories and the absence of detailed measures of disease severity may limit the ability to detect potential interaction effects.

### Limitations

Several limitations related to the matching strategy and use of registry data should be acknowledged. Comorbidities were predefined using seven broad diagnostic categories (cancer, diabetes, pulmonary disease, stroke and dementia, liver disease, kidney disease, and cardiovascular disease). This approach does not account for disease severity, duration, or overall multimorbidity burden, and individuals classified within the same category may, therefore, differ substantially in prognosis (e.g., atrial fibrillation versus congestive heart failure within the cardiovascular disease category). Consequently, substantial residual confounding is likely.

In addition, several important determinants of mortality after FNF were not captured in the registry data and could not be included in the matching or analyses. These include geriatric factors such as frailty, functional status, severity of cognitive impairment, institutionalization, and polypharmacy, as well as lifestyle-related factors such as smoking status, body mass index, alcohol consumption, and physical activity, all of which are associated with both hip fracture risk and mortality [18–21]. Furthermore, preexisting diseases may lead to an increased risk of falling (e.g., atrial fibrillation, COPD exacerbation), thereby constituting a confounding factor of death. Clinical and procedural determinants—including time to surgery, type of surgical procedure, perioperative complications, anesthesia type, and orthogeriatric co-management—were likewise unavailable. Moreover, the analyses did not distinguish between acute and chronic conditions or account for the cumulative effect of multiple coexisting diseases, which may have contributed to residual confounding.

Another limitation is that the intended 1:10 matching ratio was not achieved for all cases. Of the 3246 cases included, 486 (15%) could not be matched to 10 controls and were instead matched to a minimum of 5 controls, which may have introduced imbalance and potential selection bias.

Comorbidity status, mortality, and case–control identification were derived from the Danish National Patient Register. Although reporting to this registry has been mandatory by Danish law since 1978, and the registry has been widely used in epidemiological research with long-term follow-up, the use of administrative data is inherently limited by the available level of clinical detail [11, 12].

Furthermore, the 30-day hazard ratio of approximately 16 is notable, but it should be interpreted with caution. The very low absolute mortality in the matched reference group (0.3%) amplifies relative measures. This reflects the comparison of a major acute event—hip fracture and subsequent surgery—with a population without such an event. Our presenting of both absolute and relative risks provides a more balanced perspective on the clinical impact of hip fractures and their treatment.

Taken together, these limitations preclude attribution of excess mortality solely to the fracture itself, and the findings should, therefore, be interpreted as associations rather than causal effects. Further studies incorporating more detailed clinical and geriatric data are needed to clarify the relative contribution of specific diagnoses, multimorbidity, and functional status to mortality after hip fracture.

## Conclusion

This study demonstrated that FNFs are associated with a markedly elevated short-term mortality risk, with patients experiencing a 16 times higher risk of death within 30 days compared with an age-, gender-, and comorbidity-matched group without an FNF. The excess risk was most pronounced during the first 3–6 months and persisted for up to 4 years before converging with the match group’s mortality rates.

## Data Availability

The data are available upon reasonable request.
